# An Account of an Anencephalous Fœtus

**Published:** 1838-02-14

**Authors:** Francis West

**Affiliations:** Philadelphia


### *An Account of an Anencephalous Fœtus. By* Francis West, M. D., *of Philadelphia.*

[Illustrated with a lithograph.]

To Drs. Biddle & Clymer.

Gentlemen,—As the occurrence of a perfectly anencephalous fœtus is acknowledged by all writers upon the subject to be very rare, I have thought it might be interesting and useful to communicate to you a short history and description of a male fœtus of this kind, the product of a case of abortion at six months, which I should be glad to have recorded in the "Medical Examiner." In the following brief and imperfect sketch, I have attempted only to delineate the more characteristic features of this interesting specimen of monstrosity, leaving to others to explain the causes of their occurrence, and to fix their precise value and importance. It is a perfect specimen of what has been thought by the learned author of the article "Anencephalous," in the American Cyclopædia of Pract. Med. and Surgery, to be the rarest form of this kind of abnormal deviation, and the only one to which the term can be appropriately applied—"So seldom does it occur," he adds, "that only a few cases of it are to be found on record."—Some remarkable peculiarities of external configuration and structure exist along with the entire absence of the cerebro-spinal axis, which give to the specimen before us increased value and curiosity.

By some very essential and radical vice of formation, this human fœtus has been so materially degraded in the scale of being, as very closely to approximate, in some prominent points, the lower order of animals; and I may state that its peculiar configuration and structure would not by any possibility have permitted it to assume the erect position, supposing it capable of maintaining an independent existence. In obedience then to this necessity, which I think will be perfectly apparent from what follows, it has been represented, in the accompanying drawing, in the horizontal position, and not with the view of adding grotesqueness to its other singularities.

This anencephalous fœtus possesses all the characters belonging to the varieties, "Anencephalus" and "Derencephalus" in Geoffry St. Hilaire's classification of monsters. The cranial bones, which have been thought always to exist, though sometimes only in a rudimentary condition in fœtuses of this kind, are here *entirely absent.* The basilar process of the occipital bone is united with the bodies of the dorsal vertebræ, the intervening cervical ones having no existence; these vertebræ and those below them to the termination of the column, are "cleft posteriorly and enlarged by spina bifida, with their lateral halves much inflected outwards and separated from each other." This condition of the vertebræ leaves a large chasm in the back, about 14 lines wide, covered only by the membranous semi-circular sac, represented in the drawing.—The whole face with each individual organ of sense is much enlarged, and presents a most unnatural expression of countenance. The direction of the eyes as well as the whole face, in consequence of the excessive posterior inclination of the base of the cranium, is immediately upwards, even more so than is shown in the drawing, when the fœtus is held in the erect position, which therefore must have been attended by their total uselessness. To the whole margin of the chasm in the back, which at the angle formed by the junction of the basilar portion of the skull to the dorsal vertebræ becomes a triangular cavity of some depth, is attached the sac above mentioned, which is continued forwards on either side along the edge of the oblique plane formed by the base of the cranium and the bones of the face.

This sac which was filled with fluid was ruptured during labor; it enclosed the membranous cornua, to be seen in the drawing, and which alone occupied all the space upon which should have rested the cerebral mass. Along the margin of this bag throughout its whole extent, from the orbits to its termination at the sacrum, is an abundant growth of very dark hair, at some points more than half an inch long,—which arrangement gives the idea of the scalp having been drawn down over the back, and countenances the notion that the head with its contents or something answering to them, were to have been developed upon the back, which displays to all appearance the attempt to form there a lodgment for them.

The above impression is very strongly forced upon us by a posterior view of these parts as they exist in the preparation, which could not be given in the drawing. Portions of the membranes of the medulla spinalis, forming elongated circular sacs, containing a little thin fluid, existed upon and in close contact with the depression along the bodies of the vertebræ.

The upper and lower extremities present remarkable peculiarities which deserve especial attention in our observations and reflections upon the character and destination of this much deformed being. The clavicles do not exist at all; and the scapulæ in actual contact with the sides of the face, are attached to the fore-part and sides of the thorax, instead of posteriorly, with their long diameters perpendicular to, instead of parallel with the axis of the body; the arms and fore-arms are of unusual length and very loosely articulated at the carpo-radial articulation; the deltoid muscles are extraordinarily developed, and the skin of these, as well as that of the lower extremities has much hair growing upon it; the lower extremities are also very long and muscular, and present the same peculiarity of direction as the upper ones at their union with the body. The articulations at the ankles are very loose and admit without the least violence the touching of the metatarsus and the spine of the tibia as the foot rests upon a plane surface.*

The nerves of the extremities are fully developed, and ramify through the parts to which they are respectively sent. On tracing up these nerves they were found suddenly to terminate at the vertebræ and had no connexion with the spinal membranes spoken of.

This fact is of importance to those who contend that the nerves are formed at the periphery of the body and are developed towards the central masses, with which they afterwards unite. One or two ganglions of the sympathetic nerve were discovered in the thorax, and its dissection was not further pursued. The umbilical cord is about 1½ inches in diameter and contains the entire liver, which is closely adherent to its sides, with a large portion of the great and small intestines. The other organs of the abdomen are natural and in situ, and so are those contained in the thoracic cavity.

It was desired to pursue particularly the dissection of the nerves of animal life, but as this would materially have destroyed the preparation, the examination was reluctantly given up, and it is hoped without the sacrifice of much information.

The parents are natives of Lincolnshire, England, and were married in June last, exactly six months before the woman aborted with this monstrous fœtus. It was feared to make any inquiries of the parents in regard to the occurrence or non-occurrence of similar births in either of their families, as this must have led to suspicion about the character of the present one. The father is about 25, and the mother 28 years of age; they are perfectly healthy and well formed. They arrived at a hotel in this city much fatigued by a forced journey which they had made from Cincinnati, and the mother was very soon after taken sick. I reached her just after the waters had been discharged, and found, on examination, the chin of the child presenting at the inferior strait; a very few pains sufficed to deliver it. The umbilical cord and placenta were much diseased, and of the latter small pieces continued to come away for several days, producing each time alarming hemorrhage, which jeoparded the life of the woman. She ultimately, however, recovered perfectly and left the city.

Very respectfully, yours, &c.

Francis West, Jr.

*Philadelphia, Feb.* 2, 1838.

P.S. Since writing the above I have received a copy of the London Lancet for Nov. 4, 1837, in which I find an account of a human monstrosity, which possessed neither brain nor spinal marrow, copied from a Southern Journal of our own country. It will be observed that the case of which I have given you a brief and imperfect sketch, possesses some characters of considerable interest which do not belong to the "anencephale," of which so full a description has been given by Drs. Nicoll and Arnold, it being solely remarkable from the deficiency of the cerebro-spinal axis, which was not attended with any peculiar deformity of the child beyond that necessarily arising from the want of the cerebro-spinal mass and its investments.

* Whole length of fœtus from heel to base of cranium, 11 inches.

| | |
|---|---|
| from anus to base of cranium | 5 " |
| " external malleolus to trochanter | 6 " |
| length of femur | 3 & 6 lines |
| " tibia | 2 & 9. |
| " of foot | 2. & 3. |
| length of whole arm | 8. |
| " " humerus | 3. & 3. |
| " " fore-arm | 4. & 9. |

